# Supplementary material for: Single molecule analysis reveals reversible and irreversible steps during spliceosome activation
Source: eLife. 2016 May 31;5:e14166. doi: 10.7554/eLife.14166 (PMC4922858; doi:10.7554/eLife.14166)
Supplement: Figure 1—source data 1. — DOI: http://dx.doi.org/10.7554/eLife.14166.004 [file elife-14166-fig1-data1.docx]

**Figure 1-Supplemental Table 2**

**Fit Parameters Describing the Distribution of Dwell Times Observed for the U1 and NTC Subcomplexes**

| **Subcomplex** | **Strain** | **[ATP] mM** | **A_1_** | **τ_1_**  **(min)** | **A_2_** | **τ_2_**  **(min)** | **A_3_** | **τ_3_**  **(min)** | **Data Source** |
| --- | --- | --- | --- | --- | --- | --- | --- | --- | --- |
| U1 | yAAH6 | 2 | 0.78±0.08 | 0.062±0.019 | 0.22 | 2.3±0.5 | * | * | Hoskins et al. 2011 |
| U1 | yAAH6 | 0.05 | 0.45±0.04 | 0.15±0.02 | 0.30±0.03 | 1.3±0.4 | 0.25±0.03 | 11±1 | This work |
| U1 | yAAH6 | 0 | 0.74±0.07 | 0.12±0.07 | 0.26 | 2.0±0.8 | * | * | Hoskins et al. 2011 |
| NTC | yAAH20 | 2 | 0.54±0.08 | 0.40±0.11 | 0.46 | 4.8±0.7 | * | * | Hoskins et al. 2011 |
| NTC | yAAH20 | 0.05 | 0.94±0.18 | 0.33±0.03 | 0.06±0.03 | 3.7±1.7 | * | * | This work |

*Not applicable; fit to sum of two exponential terms.
