# Supplementary material for: Single molecule analysis reveals reversible and irreversible steps during spliceosome activation
Source: eLife. 2016 May 31;5:e14166. doi: 10.7554/eLife.14166 (PMC4922858; doi:10.7554/eLife.14166)
Supplement: Figure 2—source data 1. — DOI: http://dx.doi.org/10.7554/eLife.14166.009 [file elife-14166-fig2-data1.docx]

**Figure 2-Supplemental Table 1.**

**Oligonucleotides used for generating yeast strains**

| **Name** | **Sequence** | **Use** |
| --- | --- | --- |
| ah540_prp3_fsnap | Gga ttc ttt att acg tac gct ggg tca gtt tga ttc aga gca ttt tta ttc acc tgt tca aac ggg ttc tgg tgg ttc tgg tat gga caa aga ctg cga aat gaa gcg caC | C-terminal tagging of Prp3 with fast SNAP (SNAP_f_) with a (GSG)x2 linker |
| ah504_prp3_hygnat | GCT AAA AAA ATC ATT ATT CTG GGC GAC ATA CAG TAA AAT AAT ATT TAA TAT GAA ACA AAG CGT ATC ATT TTG TAG ACA CCG ATA GAG CTC GTT TTC GAC ACT GGA TGG C | C-terminal tagging of Prp3 and selection with HygR or NatR selectable markers |
| ah522_prp3_u | CAA ATT TTA TGA GAA TTT GGT GAT GAA GAG | Confirmation of tagged Prp3 by PCR |
| ah523_prp3_d | CTC AAA ACA TCG GTA AAT ACC TAA AAC C | Confirmation of tagged Prp3 by PCR |
| ah541_Prp4_fsnap | CGA ATA ATT CTC ATT TTT TAG TGA GCG GCG GAT GGG ATA GGT CTA TCA AGC TCT GGA ATG GTT CTG GTG GTT CTG GTA TGG ACA AAG ACT GCG AAA TGA AGC GCA C | C-terminal tagging of Prp4 with fast SNAP (SNAP_f_) with a (GSG)x2 linker |
| ah506_prp4_hygnat | GGA AAT GTA TAA TTA AAT AAA TAA TGC AGT GTT AAT AAA ACG TTA GTT TCA AAA ATA CTA AAT ACA TTT CTT TAC ACA AGA GCT CGT TTT CGA CAC TGG ATG GC | C-terminal tagging of Prp4 and selection with HygR or NatR selectable markers |
| ah524_prp4_u | GTT TGG GAC ATC AGA AAA CGG GAT GAA G | Confirmation of tagged Prp4 by PCR |
| ah525_prp4_d | TGA ATG AGA AAA TTG GCT TTT TGA AAA AGG | Confirmation of tagged Prp4 by PCR |
