# Supplementary material for: Single molecule analysis reveals reversible and irreversible steps during spliceosome activation
Source: eLife. 2016 May 31;5:e14166. doi: 10.7554/eLife.14166 (PMC4922858; doi:10.7554/eLife.14166)
Supplement: Figure 2—source data 2. — DOI: http://dx.doi.org/10.7554/eLife.14166.010 [file elife-14166-fig2-data2.docx]

**Figure 2-Supplemental Table 2**

**Fit Parameters Describing the Distribution of Dwell Times Observed for the U4 Subcomplex**

| **Subcomplex** | **Strain** | **[ATP] mM** | **A_1_** | **τ_1_**  **(min)** | **A_2_** | **τ_2_**  **(min)** | **A_3_** | **τ_3_**  **(min)** |
| --- | --- | --- | --- | --- | --- | --- | --- | --- |
| U4^a^ | yAAH67 | 2 | 0.84±0.19 | 0.25±0.04 | 0.16±0.06 | 2.5±0.8 | * | * |
| U4 | yAAH67 | 0.05 | 0.56±0.12 | 0.10±0.02 | 0.18±0.03 | 1.1±1.0 | 0.26±0.06 | 13±5 |

*Not applicable; fit to sum of two exponential terms.
