# Supplementary material for: Single molecule analysis reveals reversible and irreversible steps during spliceosome activation
Source: eLife. 2016 May 31;5:e14166. doi: 10.7554/eLife.14166 (PMC4922858; doi:10.7554/eLife.14166)
Supplement: Figure 4—source data 1. — DOI: http://dx.doi.org/10.7554/eLife.14166.020 [file elife-14166-fig4-data1.docx]

**Figure 4-Supplemental Table 1**

**Fit Parameters Describing the Distribution of Dwell Times Observed for U4/U5 Complexes that Arrived and Departed Simultaneously**

| **Subcomplex** | **Strain** | **[ATP] mM** | **A_1_** | **τ_1_**  **(min)** | **A_2_** | **τ_2_**  **(min)** | **A_3_** | **τ_3_**  **(min)** |
| --- | --- | --- | --- | --- | --- | --- | --- | --- |
| U4/U5 | yAAH71 | 2 | 0.60±0.14 | 0.06±0.02 | 0.37±0.15 | 0.6±0.2 | 0.03±0.04 | 11±11 |
| U4/U5 | yAAH71 | 0.05 | * | * | 0.67±0.11 | 0.9±0.3 | 0.33±0.08 | 13±3 |

*Not applicable; fit to sum of two exponential terms.
