# Supplementary material for: Single molecule analysis reveals reversible and irreversible steps during spliceosome activation
Source: eLife. 2016 May 31;5:e14166. doi: 10.7554/eLife.14166 (PMC4922858; doi:10.7554/eLife.14166)
Supplement: Figure 5—source data 1. — DOI: http://dx.doi.org/10.7554/eLife.14166.023 [file elife-14166-fig5-data1.docx]

**Figure 5-Supplemental Table 1**

**Fit Parameters Describing the Distribution of Dwell Times Observed for U4 or U5 after Simultaneous U4/U5 Arrival and U4 Loss Preceding U5 Loss**

| **Subcomplex** | **Strain** | **[ATP] mM** | **A_1_** | **τ_1_**  **(min)** | **A_2_** | **τ_2_**  **(min)** |
| --- | --- | --- | --- | --- | --- | --- |
| U4^a^ | yAAH71 | 2 | N/A | 1.0±0.1 | N/A | 0.06±0.01 |
| U5^b^ | yAAH71 | 2 | 0.20±0.07 | 0.70±0.23 | 0.80±0.15 | 9.0±1.9 |

^a^These data were fit to a convolution function. ^b^Fitted data represents t(release,U5) minus t(release, U4). N/A indicates Not Applicable.
